# Integrin β Expression as a New Diagnostic Marker for Arteriovenous Thrombosis: A Single-Center Prospective Study

**DOI:** 10.3390/jcdd10110461

**Published:** 2023-11-15

**Authors:** Yan Xi, Yu Mao, Fan Yang, Peng Xi, Wei Zhu, Yanli Song, Wenwen Yan, Xudong Liao, Lin Zhou

**Affiliations:** 1 Department of General Practice, Tongji Hospital, School of Medicine, Tongji University, Shanghai 200065, China; 2033352@tongji.edu.cn (Y.X.); z9529w@163.com (W.Z.); 2 Department of Cardiology, Tongji Hospital, Tongji University, Shanghai 200065, China; doctor.maoyu@hotmail.com (Y.M.); shtjtmt2010@163.com (P.X.); tj-yww@163.com (W.Y.); liaox@nankai.edu.cn (X.L.); 3 Department of Clinical Laboratory, Tongji Hospital, Tongji University, Shanghai 200065, China; ojr6157ws@163.com; 4 Department of Emergency Internal Medicine, Tongji Hospital, Tongji University, Shanghai 200065, China; drlily@163.com

**Keywords:** integrin, venous thromboembolism, acute myocardial infarction, interleukin 6, cytokine storm

## Abstract

Integrin β plays an important role in the pathogenesis of thrombosis and inflammation, and it may be a shared pathogenic mechanism between arterial and venous thromboses. With the goal of identifying new treatment targets for thrombotic diseases and specific diagnostic markers for venous thromboembolism (VTE), this prospective clinical study was performed to clarify the relationship between integrin and thrombosis. The levels of integrin β1–3, interleukin-6 (IL-6), and C-reactive protein were significantly higher in patients with acute myocardial infarction (AMI; *n* = 44) and acute VTE (*n* = 43) compared to healthy controls (*n* = 33). The IL-6 and integrin β1–3 levels were also significantly higher in the AMI group compared to the VTE and control groups. Logistic regression analysis identified IL-6 and integrin β1–3 levels as independent risk factors for thrombotic disease. Based on the receiver-operating characteristic curve, Youden index, sensitivity, and specificity, the diagnostic accuracy value for VTE was greater than 0.8 when integrins β1, β2, and β3 were combined. Overall, these results suggest that integrin β levels can contribute to improving the diagnosis and treatment of arteriovenous thrombosis.

## 1. Introduction

Thrombotic diseases account for over 25% of deaths worldwide. Although coronary atherosclerotic heart disease (CHD) and venous thromboembolism (VTE) are distinct diseases, recent evidence suggests a pathophysiological link. Dyslipidemia is a risk factor for arterial thrombotic events such as acute myocardial infarction (AMI); however, an increase in triglycerides and a decrease in high-density lipoprotein elevate the risk of VTE, whereas an increase in high-density lipoprotein may protect against VTE [1]. In addition, atherosclerosis and VTE share many similar risk factors, including old age, obesity, smoking, and diabetes [2]. A prospective study showed that patients with VTE, especially those with pulmonary embolism of unknown cause, have a significantly increased risk of arterial thromboembolism, including myocardial infarction, stroke, and sudden death of unknown cause [3,4]. Arterial thrombosis is mainly related to the formation of white thrombi caused by platelet aggregation, whereas VTE is mainly related to the formation of red thrombi caused by increased fibrinogen activity secondary to endogenous coagulation dysfunction. Accordingly, antiplatelet therapy is the cornerstone of the prevention and treatment of CHD, whereas anticoagulation is the key to the treatment of VTE. However, aspirin alone can also be effective for the primary and secondary prevention of VTE [5]. In primary prevention, aspirin monotherapy reduced the incidence of deep vein thrombosis and pulmonary embolism by 20% and 69%, respectively, in patients who received surgical treatment or had high-risk factors [6]. In addition, a randomized clinical trial showed that aspirin reduced the recurrence rate of VTE by 32% compared to placebo among patients with a history of VTE [5]. These studies suggest shared pathological mechanisms between arterial and venous thrombosis. 

The activation of platelets and the coagulation system are considered the main signs of thrombosis, and there is increasing recognition that inflammation and the interaction between related cells are key regulatory factors of thrombosis. Atherosclerosis is a chronic inflammatory disease of the arteries [7]. Cholesterol induces inflammasome activation via lysosomal damage, and oxidized low-density lipoprotein can activate the nucleotide-binding domain, leucine-rich-containing family, and pyrin domain-containing-3 (NLRP3), inducing the maturation of interleukin (IL)-1β and IL-18, which further aggravates chronic inflammation [8,9,10,11,12,13]. In a venous thrombosis mouse model, when blood flow is reduced and hypercoagulability is present, the reduced shearing force of blood flow leads to the activation of nuclear factor (NF)-κB and increases exposure to adhesion molecules, which triggers the recruitment of white blood cells [14]. In our previous study, bioinformatics methods were employed to analyze multiple key genes involved in the pathogenesis of pulmonary embolism, demonstrating that CCL2, CXCL10, tumor necrosis factor (TNF), Retnla, tissue factor pathway inhibitor 2 (TFPI2), and cytochrome P450 family 1 subfamily A1 (CYP1A1) play roles in the pathogenesis of pulmonary embolism, with chemokine signal transduction, the chemokine activity pathway, and inflammatory reactions all related to the occurrence of pulmonary embolism [15,16,17]. The incidence of myocardial injury, arrhythmia, acute coronary syndrome, and VTE were all found to be elevated in patients with coronavirus disease (COVID-19) early in the pandemic; this relationship was ascribed to the “cytokine storm” caused by infection with severe acute respiratory syndrome coronavirus 2, which increased the production of pro-inflammatory cytokines such as IL-1, IL-2, IL-6, IL-12, IL-18, TNF-α, and interferon (INF)-γ [18]. Thus, inflammation is likely involved in the pathogenesis of arteriovenous thrombosis and may be a shared pathophysiological process between arterial and venous thrombosis.

In this study, we aimed to detect the major inflammatory markers C-reactive protein (CRP), IL-6 (a cytokine closely related to arterial thrombosis), integrin β1, integrin β2, and integrin β3 in patients with acute arterial thrombosis (specifically AMI) and acute VTE. We then explored the inflammatory mechanism of acute arteriovenous thrombosis and investigated potentially useful diagnostic markers to provide a new direction for the diagnosis and treatment of arteriovenous thrombosis.

## 2. Materials and Methods

### 2.1. Study Population

For this prospective study, we recruited a total of 120 participants, including 43 patients with VTE (VTE group), 44 patients with AMI (AMI group), and 33 healthy controls. The diagnostic criteria for VTE (including deep venous thrombosis and acute pulmonary embolism) were as follows: (1) chest pain, dyspnea, lower limb swelling, and other clinical symptoms; (2) a positive D-dimer test; and (3) diagnosis using spiral computed tomography (CT) and Doppler ultrasonography. The diagnostic criteria for AMI (including ST-segment elevation myocardial infarction and non-ST-segment elevation myocardial infarction) were as follows: (1) increase in the serum levels of troponin, a marker of myocardial injury, to more than 99% of the upper limit of the normal value in at least one determination; (2) clinical evidence of myocardial ischemia (at least one of the following manifestations: clinical manifestations of AMI, myocardial ischemia on electrocardiogram, new pathological Q wave, and evidence of new abnormal segmental motion of the ventricular wall upon imaging examination); and (3) diagnosis based on coronary angiography. The neutrophil count, hemoglobin (Hb) level, lymphocyte count, platelet count, and levels of troponin, CRP, D-dimer, IL-6, integrin β1, integrin β2, and integrin β3 were detected in all patients. This study was approved by the Ethics Committee of Shanghai Tongji Hospital (No.: K-W-2022-032), and informed consent was obtained from each participant before the study. Our study was carried out in accordance with the ethical standards of the Declaration of Helsinki.

### 2.2. Exclusion Criteria

The exclusion criteria were as follows: (1) incomplete medical records; (2) adolescents or pregnant individuals; (3) severe liver or kidney injury or thyroid dysfunction; (4) non-coronary atherosclerotic myocardial infarction, including coronary microvascular dysfunction, coronary spasm, coronary muscle bridge, coronary inflammation, and coronary dissection; (5) COVID-19, immunosuppressive diseases, and respiratory diseases; and (6) use of anticoagulants, antiplatelets, antibiotics, or other drugs in the month prior to the study.

### 2.3. Flow Cytometry

After admission, 2 mL of peripheral venous blood was collected from individuals in the VTE, AMI, and control groups and injected into a vacuum tube containing heparin. Blood samples were collected within 24 h after the onset of the disease. For the detection of integrin, anticoagulated blood (100 μL) was incubated with phycoerythrin-conjugated anti-CD29 (integrin β1; AB_2536494, Thermo Fisher Scientific, Waltham, MA, USA), fluorescein isothiocyanate-conjugated anti-CD18 (integrin β2; AB_10698010, eBioscience, San Diego, CA, USA), and anti-CD61 (integrin β3; AB_1227581, BioLegend, San Diego, CA, USA) monoclonal antibodies in the dark at 25 °C for 30 min, followed by the addition of hemolysin (500 μL). After incubation at 37 °C for 30 min, the mixture was rinsed three times and subjected to flow cytometry on a FACS Via system (BD Company, Franklin Lanes, NJ, USA).

### 2.4. Statistical Analysis

Continuous variables are expressed as mean ± standard deviation and were compared using one-way analysis of variance among groups, followed by least-significant difference analysis for pairwise comparisons. Comparisons between groups were performed using post-hoc testing (LSD). The normality of the quantitative data was determined using the Shapiro–Wilk test. Categorical data are expressed as proportions and were compared using the chi-square test between groups. Receiver operating characteristic (ROC) curves were created, and area under the curve (AUC) values were calculated to ascertain the predictive ability of integrin β1, integrin β2, and integrin β3 in determining the diagnostic performance for acute AMI and VTE; the optimal cut-off value, area under the ROC curve (AUC), sensitivity, specificity, and other indicators were calculated. The odds ratio was used to determine the factors that influence arterial thrombosis. Multivariate logistic regression analysis was employed to evaluate the predictive value of a combination of different factors, and the prediction probability was generated for further ROC curve analysis. R software (version 4.2.2) (pROC, ggplot2 packages) was used for statistical analysis and figure generation. A two-tailed *p*-value of <0.05 was considered statistically significant.

## 3. Results

### 3.1. General Clinical Characteristics and Laboratory Testing Indicators 

A total of 120 participants were included in this study, divided into the VTE, AMI, and control groups according to their clinical diagnosis. We identified no significant differences in sex or age among the three groups (*p* > 0.05) (Table 1). 

**Figure 1:**
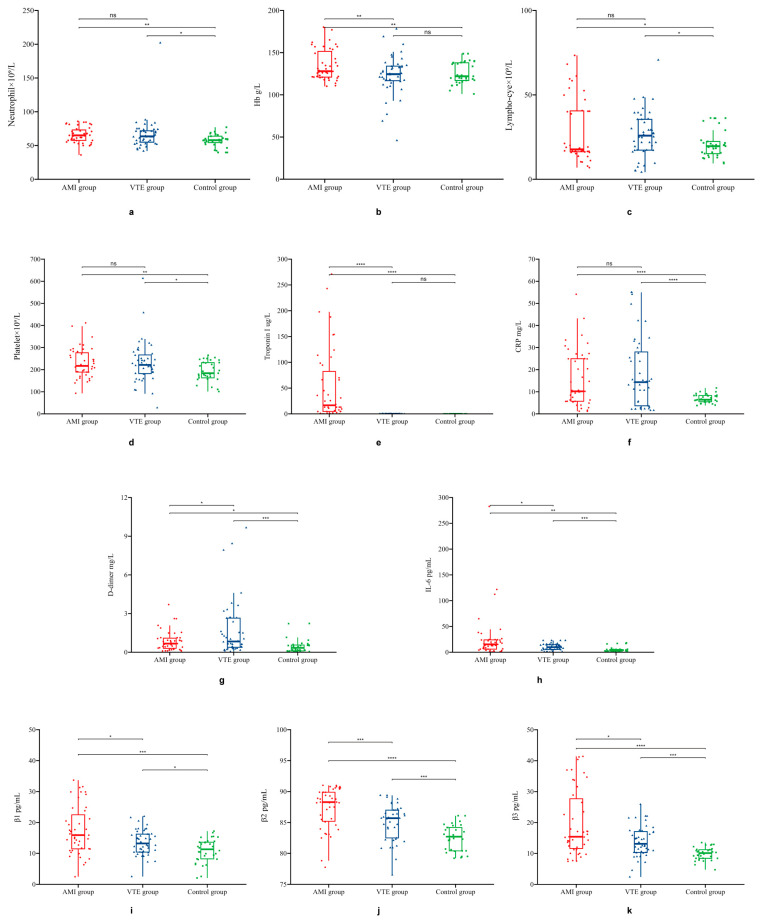
Expression levels of neutrophils, Hb, lymphocytes, platelets, troponin I, CRP, D-dimer, IL-6, integrin β1, integrin β2, and integrin β3 in the AMI, VTE, and control groups. Box-and-whisker plots showing the levels of (**a**) neutrophils, (**b**) Hb, (**c**) lymphocytes, (**d**) platelets, (**e**) troponin I, (**f**) CRP, (**g**) D-dimer, (**h**) IL-6, (**i**) integrin β1, (**j**) integrin β2, and (**k**) integrin β3 in the AMI, VTE, and control groups. VTE, venous thromboembolism; AMI, acute myocardial infarction. * *p* < 0.05, ** *p* < 0.01, *** *p* < 0.001, **** *p* < 0.0001. ns, no statistical significance.

The VTE group exhibited significantly increased levels of CRP, D-dimer, IL-6, integrin β1, integrin β2, and integrin β3 when compared with the control group (Figure 1). 

Similarly, compared with those of the control group, the AMI group exhibited significantly increased levels of Hb, troponin I, CRP, D-dimer, IL-6, integrin β1, integrin β2, and integrin β3 (*p* < 0.05). The levels of Hb, troponin I, IL-6, integrin β1, integrin β2, and integrin β3 were also significantly higher in the AMI group than in the VTE group (*p* < 0.05). However, D-dimer and CRP levels were significantly higher in the VTE group than in the AMI group. Neutrophil, lymphocyte, and platelet counts were significantly higher in the AMI and VTE groups than in the control group (*p* < 0.05) but did not differ significantly between the AMI and VTE groups (Table 1). 

### 3.2. Factors Influencing Arterial Thrombosis and Diagnostic Performance in AMI 

In the AMI group, the neutrophil, lymphocyte, and platelet counts and Hb, CRP, D-dimer, IL-6, integrin β1, integrin β2, and integrin β3 levels were included in the univariate logistic regression equations. The results showed that neutrophil and lymphocyte counts, Hb, CRP, IL-6, integrin β1, integrin β2, and integrin β3 levels were independent predictors of arterial thrombosis (*p* < 0.05). The lowest *p* values (≤0.001) were observed for IL-6, integrin β1, integrin β2, and integrin β3, thus their significant impact on arterial thrombosis (Table 2). ROC curve analysis showed high diagnostic sensitivity and specificity of integrin β1, integrin β2, and integrin β3 for AMI with cut-off values of 15.7 ng/mL, 86.15 ng/mL, and 13.55 ng/mL and corresponding AUC values of 0.751, 0.859, and 0.830, respectively (Table 3).

**Figure 2:**
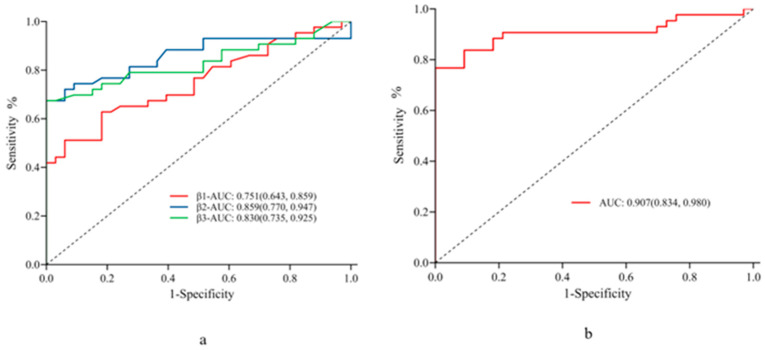
Diagnostic performance of integrin β1, integrin β2, and integrin β3 for arterial thrombosis. Receiver operating characteristic curves showing the diagnostic performance (AUC) of different markers for arterial thrombosis: (**a**) integrin β1, integrin β2, and integrin β3, and (**b**) the combination of all three markers. AUC: area under the ROC curve.

The combination of integrin β1, integrin β2, and integrin β3 resulted in an improved AUC value of 0.907 for the diagnosis of AMI compared to either marker alone (Figure 2).

### 3.3. Factors Influencing Venous Thrombosis and Diagnostic Performance in VTE 

The same variables were included in the univariate logistic regression equation for VTE as used for AMI. The results showed that the neutrophil and lymphocyte count and CRP, D-dimer, IL-6, integrin β1, integrin β2, and integrin β3 levels were all independent predictors of venous thrombosis. The levels of D-dimer, IL-6, integrin β1, integrin β2, and integrin β3 significantly impacted venous thrombosis (Table 4). 

According to the ROC curve constructed using clinical diagnosis as the criterion, the diagnostic sensitivity and specificity for venous thrombosis were high for integrin β1, integrin β2, and integrin β3 at cut-off values of 14.3 ng/mL, 85.4 ng/mL, and 13.55 ng/mL with corresponding AUC values of 0.666, 0.722, and 0.758, respectively; the AUC value for the combination of the three markers increased to 0.802; D-2 showed an AUC value of 0.758 (Table 5). 

**Figure 3:**
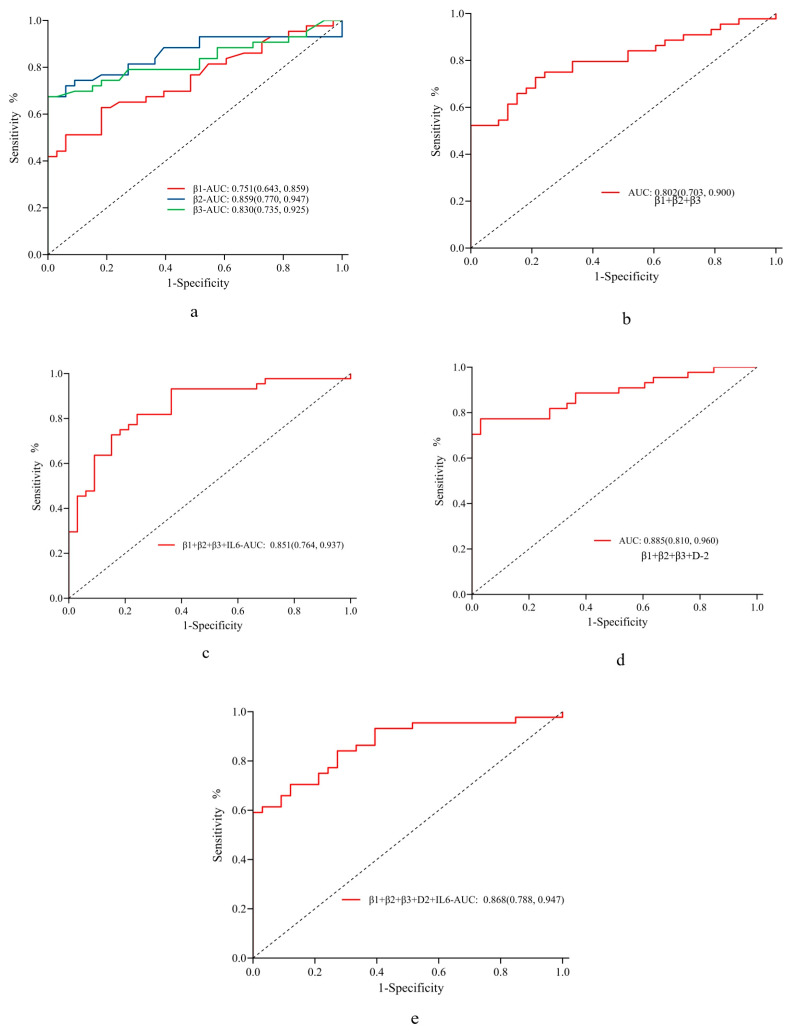
Diagnostic performance of IL-6, integrin β1, integrin β2, and integrin β3 for arterial thrombosis. Receiver operating characteristic curves showing the diagnostic performance (AUC) of different markers for venous thrombosis: (**a**) integrin β1, integrin β2, and integrin β3; (**b**) all three integrins combined; (**c**) all three integrins combined with IL-6; (**d**) all three integrins combined with D-dimer; and (**e**) all three integrins combined with D-dimer and IL-6. AUC, area under the ROC curve; IL, interleukin.

Furthermore, the AUC value of the three integrins combined with IL-6 was 0.851, that of the three integrins combined with D-dimer was 0.866, and that of the three integrins combined with D-dimer and IL-6 was 0.8, all of which suggested favorable diagnostic performance (Figure 3). 

## 4. Discussion

In this study, we detected major inflammatory markers (CRP and IL-6), integrin β1, integrin β2, and integrin β3 in patients with AMI and VTE, as well as in healthy controls. Our results showed that the levels of CRP, IL-6, integrin β1, integrin β2, and integrin β3 were significantly higher in the AMI and VTE groups than in the control group, suggesting that the pathogenesis of arteriovenous thrombosis is closely related to the inflammatory status. In acute inflammation, IL-6 can induce the synthesis of CRP [19]. Our study indicated that IL-6 expression was 5 times higher in the AMI group than in the control group and was 2.5 times higher than in the VTE group (*p* < 0.05). The CRP content was also higher in the AMI and VTE groups than in the control group, which is consistent with our previous findings that the hs-CRP content is significantly increased in patients with CHD and VTE, suggesting that acute arteriovenous thrombosis is closely related to inflammatory hyperactivation [20]. Inflammation-induced coagulation activation is an important mechanism of host defense against pathogens. Under these conditions, the interaction between innate immunity and platelets limits the transmission of pathogens in the circulation [21]. However, when excessive inflammation is present, the activation of abnormal coagulation triggers acute thrombotic events, including AMI, stroke, and acute VTE secondary to the rupture of atherosclerotic plaques [22]. Ziltivekimab is an IL-6 ligand inhibitor and a promising new cardiovascular protective drug; however, its role in the prevention and treatment of VTE requires further research [13]. 

Recent research on the relationship between inflammation and arterial thrombosis has indicated that integrins play a role in the pathogenesis of acute arterial thrombotic events caused by excessive inflammation, from early inflammation induction to late acute thrombotic events [21]. In the present study, the expression of integrin β1, integrin β2, and integrin β3 significantly increased in the AMI group, which is consistent with previous findings. The expression of integrin β1, integrin β2, and integrin β3 also increased significantly in the VTE group, but to a slightly lower degree than that in the AMI group. Interestingly, integrin β1 is related to lymphocyte activation [23]. Although the results of our study suggest that integrins β1, β2, and β3 are involved in the occurrence and development of VTE, the specific mechanism remains unclear.

In our previous study, a human genome microarray combined with fluorescence quantitative polymerase chain reaction was used to detect mRNA levels in the peripheral blood cells of patients with VTE [16]. The results showed that the expression level of T cell-related mRNA changed significantly in the VTE group, and this phenomenon was accompanied by a Th1/Th2 cell imbalance. In patients with VTE, the cluster of differentiation markers of T lymphocytes becomes disordered [24]. Fibrinogen is the main component of venous thrombi, and some studies have found that integrins β2 and β3 bind to fibrinogen in the early stage of venous thrombosis [23]. Integrin β2 expression is a marker of leukocyte activation, and its binding to ligands such as fibrinogen is involved in inflammation and thrombosis [20]. Moreover, the binding of integrin β3, which is indicative of platelet activation, to ligands such as fibrinogen may cause platelet aggregation and thrombosis [23]. Our results indicate that integrin β1, integrin β2, and integrin β3 are involved in the occurrence and development of VTE.

As an adhesion molecule, integrin plays a role in the signaling pathway of inflammation and may be a potential target in antithrombotic therapy [23]. A previous study reported that integrin Mac-1 (aMβ2) is involved in leukocyte recruitment and platelet adhesion, and the GPIba binding site and integrin Mac-1 play important roles in thrombosis [25]. Therefore, targeting these sites could reduce the risk of thrombosis without increasing the risk of bleeding. The signal transduction of integrin requires proteins, and Kind3 is a binding protein that is crucial for the activation and adhesion of integrin β1 and integrin β3 [21]. However, Kind3−/− platelets can fail to aggregate; thus, integrin β1 and integrin β3 may serve as targets in the treatment of venous thrombosis [24]. Currently, antithrombotic therapy is still based on traditional antiplatelet and anticoagulation agents; however, these treatments increase the risk of bleeding and related mortality [6]. Therefore, the targeted inhibition of inflammation may provide a new direction for the prevention and treatment of acute arteriovenous thrombosis [23]. 

In clinical practice, deep venous thrombosis lacks highly specific markers when compared to arterial thrombosis, which results in high rates of misdiagnosis and missed diagnoses. D-dimer is the most commonly used parameter in the diagnosis of VTE, with a negative predictive value of approximately 100%. However, D-dimer is not an ideal diagnostic indicator for VTE owing to its low specificity (approximately 40%). In addition, D-dimer content increases with age, and false-positive test results are frequently reported in older patients, which further reduces the specificity of D-dimer in the diagnosis of VTE in these populations [26]. Performing imaging examinations for all patients with suspected VTE is impractical. Thus, it is imperative to develop new diagnostic markers for VTE, as attempted in this study. Our results show that the combination of integrin β1, integrin β2, and integrin β3 can achieve better diagnostic performance for VTE than D-dimer, with respective AUC values of 0.802 and 0.758. In addition, the AUC values for all three integrins combined with D-dimer or both IL-6 and D-dimer were 0.866 and 0.868, respectively, indicating even higher diagnostic efficiency for VTE. These new diagnostic markers exhibit potential for the rapid and accurate clinical diagnosis of VTE, as well as a reduction in the rates of misdiagnosis and missed diagnosis.

This study has several limitations. First, this was a single-center controlled study that included only 120 Chinese patients, representing a small sample size and a single ethnic group. Thus, future research should aim to increase the sample size and include multiple centers and ethnicities to validate our conclusions. Second, we were unable to fully evaluate integrin β. Specifically, further prospective cohort studies are required to confirm the causal relationship between subunit expression levels and acute arteriovenous disease. Third, the testing time differed among the patients enrolled in this study, and plasma could not be obtained at the same time point. In addition, the half-life and optimal testing period of various inflammatory factors may be different, and the plasma isolated from peripheral blood may not fully reflect the immune tissue reactions. Fourth, we did not determine differences in the subunit expression levels of integrin β before and after acute arteriovenous disease, which is an important limitation of this research. Lastly, the results of the ROC analysis are relative; therefore, it is necessary to carefully explain the cut-off values and the predicted values of integrin β subtypes. 

## 5. Conclusions

The levels of CRP, IL-6, and integrins β1, β2, and β3 in patients with acute arteriovenous thrombosis were higher than those in the control group, suggesting that inflammation is involved in acute arteriovenous thrombosis and may represent a shared pathway between arterial and venous thrombosis. Compared to those of the control and VTE groups, the AMI group exhibited significantly higher levels of IL-6, integrin β1, integrin β2, and integrin β3, suggesting a higher inflammatory response. Thus, targeted anti-inflammation therapy may represent a new direction for the prevention and treatment of acute arteriovenous thrombosis. Furthermore, integrin β1, integrin β2, and integrin β3 combined with IL-6 exhibited better diagnostic performance for VTE than the traditional diagnostic marker D-dimer.

## Figures and Tables

**Table 1:** Clinical characteristics of individuals in different groups.

	Total *n* = 120	AMI Group *n* = 43	VTE Group *n* = 44	Control Group *n* = 33	*p* (Overall)	*p* (AMI vs. VTE)	*p* (AMI vs. Control)	*p* (VTE vs. Control)
Age	66.84 ± 11.94	65.21 ± 12.29	67.07 ± 13.22	68.67 ± 9.51	0.455	0.499	0.171	0.539
Number of men (%)	59 (49.17%)	25 (58.14%)	20 (45.45%)	14 (42.42%)	0.328	0.332	0.260	0.974
Neutrophil	63.72 ± 17.15	65.40 ± 11.82	66.86 ± 23.84	57.37 ± 9.39	0.039	0.717	0.001	0.019
Hb	128.12 ± 19.80	135.14 ± 18.27	123.14 ± 23.58	125.61 ± 12.87	0.012	0.009	0.009	0.559
Lymphocyte	25.70 ± 15.19	28.75 ± 19.63	26.34 ± 13.62	20.87 ± 8.09	0.075	0.509	0.020	0.031
Platelet	219.31 ± 76.06	229.40 ± 67.81	231.11 ± 95.06	190.42 ± 46.38	0.036	0.923	0.004	0.016
Troponin I	21.00 ± 51.32	53.71 ± 70.98	0.02 ± 0.04	0.01 ± 0.01	<0.001	<0.001	<0.001	0.265
CRP	14.22 ± 13.14	15.78 ± 12.61	18.15 ± 16.15	6.93 ± 1.90	<0.001	0.446	<0.001	<0.001
D-dimer	1.08 ± 1.54	0.86 ± 0.77	1.76 ± 2.23	0.46 ± 0.53	<0.001	0.014	0.011	0.001
IL-6	14.58 ± 29.92	26.51 ± 47.30	10.10 ± 6.46	5.00 ± 4.81	0.003	0.029	0.005	<0.001
Integrin β1	14.20 ± 6.25	17.38 ± 7.93	13.53 ± 4.14	10.96 ± 3.84	<0.001	0.006	<0.001	0.006
Integrin β2	84.70 ± 4.12	86.98 ± 4.07	84.17 ± 4.26	82.44 ± 2.08	<0.001	0.002	<0.001	0.021
Integrin β3	14.84 ± 8.14	19.70 ± 10.69	13.80 ± 4.99	9.90 ± 2.05	<0.001	0.002	<0.001	<0.001

Data are presented as mean ± standard deviation or number (%) as relevant. Abbreviations: VTE, venous thromboembolism; AMI, acute myocardial infarction; IL, interleukin; CRP, C-reactive protein; Hb, hemoglobin.

**Table 2:** Binary logistic regression analysis of factors influencing arterial thrombosis.

Variable	OR (95% CI)	*p*-Value
Hb	1.040 (1.009, 1.077)	0.018
Neutrophil	1.073 (1.026, 1.132)	0.004
Lymphocyte	0.938(0.891, 0.982)	0.009
Platelet	1.012 (1.004, 1.022)	0.010
CRP	1.171 (1.074, 1.330)	0.003
D-dimer	2.952 (1.282, 8.629)	0.025
IL-6	1.169 (1.083, 1.288)	<0.001
Integrin β1	1.205 (1.094, 1.366)	0.001
Integrin β2	1.485 (1.257, 1.826)	<0.001
Integrin β3	1.477 (1.221, 1.892)	<0.001

Abbreviations: IL, interleukin; CRP, C-reactive protein; Hb, hemoglobin; OR, odds ratio; CI, confidence interval.

**Table 3:** Diagnostic value of integrin β1, β2, and β3 in arterial thrombosis.

Variable	Cut-Off Value	AUC (95% CI)	*p*-Value	Sensitivity (95% CI)	Specificity (95% CI)	Accuracy (95% CI)	Positive Predictive Value (95% CI)	Negative Predictive Value (95% CI)
Integrin β1	15.700	0.751 (0.643, 0.859)	<0.001	0.512 (0.362, 0.661)	0.939 (0.858, 1.000)	0.697 (0.692, 0.703)	0.917 (0.806, 1.027)	0.596 (0.463, 0.730)
Integrin β2	86.150	0.859 (0.770, 0.947)	<0.001	0.674 (0.534, 0.814)	1.000 (1.000, 1.000)	0.816 (0.812, 0.820)	1.000 (1.000, 1.000)	0.702 (0.571, 0.833)
Integrin β3	13.550	0.830 (0.735, 0.925)	<0.001	0.674 (0.534, 0.814)	1.000 (1.000, 1.000)	0.816 (0.812, 0.820)	1.000 (1.000, 1.000)	0.702 (0.571, 0.833)

Abbreviations: CI, confidence interval; AUC, area under the receiver operating characteristic curve.

**Table 4:** Binary logistic regression analysis of factors influencing venous thrombosis.

Variables	OR (95% CI)	*p*-Value
Hb	0.993 (0.969, 1.017)	0.584
Neutrophil	1.058 (1.013, 1.111)	0.017
Lymphocyte	0.960 (0.919, 0.998)	0.051
Platelet	1.008 (1.001, 1.017)	0.034
CRP	1.138 (1.059, 1.262)	0.004
D-dimer	3.187 (1.604, 8.471)	0.006
IL-6	1.176 (1.075, 1.309)	0.001
Integrin β1	1.178 (1.046, 1.348)	0.010
Integrin β2	1.156 (1.012, 1.345)	0.042
Integrin β3	1.336 (1.150, 1.617)	0.001

Abbreviations: IL, interleukin; CRP, C-reactive protein; Hb, hemoglobin; OR, odds ratio; CI, confidence interval.

**Table 5:** Diagnostic performance of integrin β1, integrin β2, integrin β3, and D-dimer in venous thrombosis.

Variable	Cut-Off Value	AUC (95% CI)	*p*-Value	Sensitivity (95% CI)	Specificity (95% CI)	Accuracy (95% CI)	Positive Predictive Value (95% CI)	Negative Predictive Value (95% CI)
D-2	0.605	0.758 (0.651, 0.864)	<0.001	0.636 (0.494, 0.779)	0.788 (0.648, 0.927)	0.701 (0.696, 0.707)	0.800 (0.667, 0.933)	0.619 (0.472, 0.766)
β1	14.300	0.666 (0.546, 0.787)	0.007	0.477 (0.330, 0.625)	0.818 (0.687, 0.950)	0.623 (0.617, 0.629)	0.778 (0.621, 0.935)	0.540 (0.402, 0.678)
β2	85.400	0.722 (0.606, 0.838)	<0.001	0.545 (0.398, 0.693)	0.939 (0.858, 1.000)	0.714 (0.709, 0.719)	0.923 (0.821, 1.026)	0.608 (0.474, 0.742)
β3	13.550	0.758 (0.650, 0.866)	<0.001	0.500 (0.352, 0.648)	1.000 (1.000, 1.000)	0.714 (0.709, 0.719)	1.000 (1.000, 1.000)	0.600 (0.471, 0.729)

CI, confidence interval; AUC, area under the receiver operating characteristic curve.

## Data Availability

The datasets generated during and/or analyzed during the current study are not publicly available due to patient privacy but are available from the corresponding author on reasonable request.
